# Supplementary material for: Fast in-vitro screening of FLT3-ITD inhibitors using silkworm-baculovirus protein expression system
Source: PLoS One. 2022 May 5;17(5):e0261699. doi: 10.1371/journal.pone.0261699 (PMC9070948; doi:10.1371/journal.pone.0261699)
Supplement: S1 File — (DOCX) [file pone.0261699.s001.docx]

Supporting Information

Fast *in-vitro* screening of FLT3-ITD inhibitors using silkworm-baculovirus protein expression system

Naoki Yamamoto^1*^, Jiro Kikuchi^2^, Yusuke Furukawa^2^, Naoya Shibayama^1*^

^1^Division of Biophysics, Department of Physiology, School of Medicine, Jichi Medical University, 3311-1 Yakushiji, Shimotsuke, Tochigi 329-0498, Japan

^2^Division of Stem Cell Regulation, Center for Molecular Medicine, School of Medicine, Jichi Medical University, 3311-1 Yakushiji, Shimotsuke, Tochigi 329-0498, Japan

*Corresponding authors; N.Y. (nyamamoto@jichi.ac.jp), N.S. (shibayam@jichi.ac.jp)

The amino acid sequence of the FLT3-ITD protein expressed in this study. The red and yellow parts indicate the duplicated and template amino acid sequences, respectively. The sequence highlighted by gray is an additional part including the FLAG tag sequence, DYKDDDK..

MHKYKKQFRYESQLQMVQVTGSSDNEYFYVDFPQYFYVDFREYEYDLKWEFPRENLEFGKVLGSGAFGKVMNATAYGISKTGVSIQVAVKMLKEKADSSEREALMSELKMMTQLGSHENIVNLLGACTLSGPIYLIFEYCCYGDLLNYLRSKREKFSEDEIEYENQKRLEEEEDLNVLTFEDLLCFAYQVAKGMEFLEFKSCVHRDLAARNVLVTHGKVVKICDFGLARDIMSDSNYVVRGNARLPVKWMAPESLFEGIYTIKSDVWSYGILLWEIFSLGVNPYPGIPVDANFYKLIQNGFKMDQPFYATEEIYIIMQSCWAFDSRKRPSFPNLTSFLGCQLADAEEAMYQNVSRLVPRGSGSGSGLRMGGSDYKDDDDK

The DNA sequence of FLT3-ITD protein inserted to the pM23 vector

ATGCACAAGTACAAAAAGCAATTTAGGTATGAAAGCCAGCTACAGATGGTACAGGTGACCGGCTCCTCAGATAATGAGTACTTCTACGTTGATTTCCCCCAGTACTTCTACGTTGATTTCAGAGAATATGAATATGATCTCAAATGGGAGTTTCCAAGAGAAAATTTAGAGTTTGGGAAGGTACTAGGATCAGGTGCTTTTGGAAAAGTGATGAACGCAACAGCTTATGGAATTAGCAAAACAGGAGTCTCAATCCAGGTTGCCGTCAAAATGCTGAAAGAAAAAGCAGACAGCTCTGAAAGAGAGGCACTCATGTCAGAACTCAAGATGATGACCCAGCTGGGAAGCCACGAGAATATTGTGAACCTGCTGGGGGCGTGCACACTGTCAGGACCAATTTACTTGATTTTTGAATACTGTTGCTATGGTGATCTTCTCAACTATCTAAGAAGTAAAAGAGAAAAATTTTCTGAAGATGAAATTGAATATGAAAACCAAAAAAGGCTGGAAGAAGAGGAGGACTTGAATGTGCTTACATTTGAAGATCTTCTTTGCTTTGCATATCAAGTTGCCAAAGGAATGGAATTTCTGGAATTTAAGTCGTGTGTTCACAGAGACCTGGCGGCCAGGAACGTGCTTGTCACCCACGGGAAAGTGGTGAAGATATGTGACTTTGGATTGGCTCGAGATATCATGAGTGATTCCAACTATGTTGTCAGGGGCAATGCCCGTCTGCCTGTAAAATGGATGGCCCCCGAAAGCCTGTTTGAAGGCATCTACACCATTAAGAGTGATGTCTGGTCCTACGGAATATTACTGTGGGAAATCTTCTCACTTGGTGTGAATCCTTACCCTGGCATTCCGGTTGATGCTAACTTCTACAAACTGATTCAAAATGGATTTAAAATGGATCAGCCATTTTATGCTACAGAAGAAATATACATTATAATGCAATCCTGCTGGGCTTTTGACTCAAGGAAACGGCCATCCTTCCCTAATTTGACTTCGTTTTTAGGATGTCAGCTGGCAGATGCAGAAGAAGCGATGTATCAGAATGTGTCTAGACTGGTTCCGCGTGGATCCGGCTCTGGATCTGGCCTCAGGATGGGGGGTTCTGACTACAAGGACGATGACGACAAGTAG

Minimal data set for Fig.1C

Minimal data set for Fig.1D

Minimal data set for Fig.2A

Minimal data set for Fig.2B
