# Supplementary figures and images for: Fast in-vitro screening of FLT3-ITD inhibitors using silkworm-baculovirus protein expression system
Source: PLoS One. 2022 May 5;17(5):e0261699. doi: 10.1371/journal.pone.0261699 (PMC9070948; doi:10.1371/journal.pone.0261699)

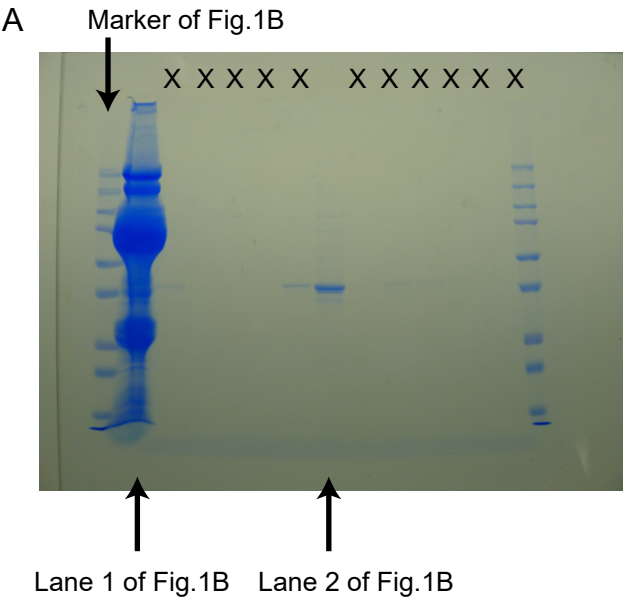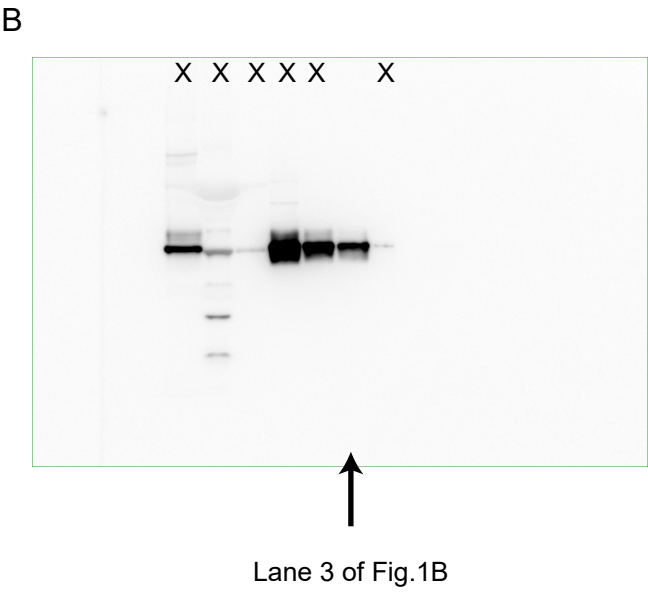

Supplement: S1 Raw images — (PDF) [file pone.0261699.s002.pdf]
