# Supplementary material for: Fast in-vitro screening of FLT3-ITD inhibitors using silkworm-baculovirus protein expression system
Source: PLoS One. 2022 May 5;17(5):e0261699. doi: 10.1371/journal.pone.0261699 (PMC9070948; doi:10.1371/journal.pone.0261699)
Supplement: Attachment — Submitted filename: Response_to_reviewers_PlosOne_220222.docx [file pone.0261699.s003.docx]

Response to the reviewers’ comments

(The yellow highlighted parts are those modified in the main text)

*Reviewer #1: In the era of personalized medicine, it becomes important to have effective and fast techniques for the detection of active molecules. This work clearly and rigorously presents a useful and fast system for this type of activity. The point that could be developed in the future is the study of the different lengths of the ITD on the efficacy of the inhibition.*

Reply

We appreciate that the importance of our study is well understood. As the reviewer mentioned, we would like to deal with FLT-ITD proteins with different ITD lengths, so that new drugs optimized for the personalized medicine will be efficiently found.

*Reviewer #2: Major comments：*

*1. To date, there have been some commercially-available FLT3-ITD proteins by which the kinase activity can be evaluated. Why are you still doing this work? please provide evidence if you purified protein better than the commercial one.*

Reply

Thank you for giving a comment discussing about the importance of our study. We would like to answer them by the point-by-point manner.

As the reviewer mentioned, there has already been commercially-available FLT3-ITDs. For example, SignalChem supplies a FLT3-ITD protein (https://signalchem.com/product_details.php?id=ZWE1ZDJmMWM0NjA4MjMyZTA3ZDNhYTNkOTk4ZTUxMzUtMTE5MDM=#specifications). However, the purity of the protein is low because it contains non-negligible contaminated proteins around 26 kDa (Figure R1). On the other hand, in our case the FLT3-ITD protein appears as the major band in the SDS-PAGE as shown in Figure 1B, which represents that we purified protein better than the commercial case.

Figure R1. SDS-PAGE of a commercially-available FLT-ITD protein produced by SignalChem. The picture was cited from a document P1749-6.pdf which can be downloaded from the website that is mentioned in the text.

2. In the abstract， “We performed an inhibition assay using 17 potential kinase inhibitors, and succeeded in identifying two potent inhibitors for FLT3-ITD.” It is easy to mislead readers into thinking that you have found two new inhibitors.

Reply

Following what the reviewer suggested, we have changed the corresponding sentence as follows;

(Page 2, line 6)

We performed an inhibition assay using 17 kinase inhibitors, and succeeded in screening two inhibitors for FLT3-ITD.

In addition to this, one sentence in conclusion was also modified as follows;

(page 10, line 3)

As far as we know, it is the first time to successfully express and purify soluble FLT3-ITD protein.

(Original sentence; As far as we know, it is the first time to successfully express a soluble FLT3-ITD protein.)

*Minor comments：*

*Some details of the materials and methods need to be supplemented, the following are specific examples:*

*1. What is the template for ITD mutation sequence PCR?*

Reply

We added the DNA sequence confirmed in the pM23 vector in Supporting Information.

*2. When co-transfected pM23 vector with linearized BmNPV DNA, what is the transfection reagent and what is the amount of transfection system? And what are the cell culture conditions?*

Reply

The transfection reagent and its amount are not open because such information is protected under the patent of ProCube system owned by Sysmex Corporation. The cell culture was harvested at 25 ºC. We added this information in Materials and Methods.

*3. Obviously, not all of the homologous recombination between the two DNA can successfully occurs, how did you screen that?*

We did not confirm if each cell was infected. However, we were able to tell that 80-90 % of the cells were infected by the virus judging from their appearance.

*4. What is amount of the recombinant virus used for injection? And after injection, what conditions are the pupae placed in?*

Reply

50 μl of the virus at 10^5^～10^6^ PFU/ml was used for the infection. The infected pupae were put at 25 ºC and 50 % humidity. We added these experimental conditions in Materials and Methods.

*Next are some of my questions or suggestions:*

*1. During the 7 days incubation period after transfection, I would like to know if you change the medium for the old cells or collect the recombinant virus to re-infect the new cells?*

Reply

We did not change the medium.

*2. Fig 1. (B) Lane 1 and Lane 2 appear to be two images stitched together; and I think adding one lane, the supernatant of the normal pupae (blank control) would be better.*

Reply

Since we do not find the merit to add the supernatant of the normal pupae to the SDS-PAGE result, we do not include it.

*3. In the Results and discussion, 3.1, SDS PAGE missed a “-”.*

Reply

We added the missing hyphen at the corresponding place.

*4. In conclusion, “Thus, the present expression system using the silkworm pupae is quite effective way to obtain the active FLT3-ITD protein”, before “quite” need to add “a”.*

Reply

We added the missing word at the corresponding place.

*5. The references may be a little less, and mostly not up to date.*

Reply

We added some up-to-date references in Introduction.
